# Cold Vapor Generation beyond the Input Solar Energy Limit

**DOI:** 10.1002/advs.201800222

**Published:** 2018-05-03

**Authors:** Haomin Song, Youhai Liu, Zhejun Liu, Matthew H. Singer, Chenyu Li, Alec R. Cheney, Dengxin Ji, Lyu Zhou, Nan Zhang, Xie Zeng, Zongmin Bei, Zongfu Yu, Suhua Jiang, Qiaoqiang Gan

**Affiliations:** ^1^ Department of Electrical Engineering The State University of New York at Buffalo Buffalo NY 14260 USA; ^2^ Material Science Department Fudan University Shanghai 200433 China; ^3^ Department of Electrical and Computer Engineering University of Wisconsin Madison WI 53705 USA

**Keywords:** cold vapor generation, perfect energy conversion, solar still, solar–thermal conversion, water purification

## Abstract

100% efficiency is the ultimate goal for all energy harvesting and conversion applications. However, no energy conversion process is reported to reach this ideal limit before. Here, an example with near perfect energy conversion efficiency in the process of solar vapor generation below room temperature is reported. Remarkably, when the operational temperature of the system is below that of the surroundings (i.e., under low density solar illumination), the total vapor generation rate is higher than the upper limit that can be produced by the input solar energy because of extra energy taken from the warmer environment. Experimental results are provided to validate this intriguing strategy under 1 sun illumination. The best measured rate is ≈2.20 kg m^−2^ h^−1^ under 1 sun illumination, well beyond its corresponding upper limit of 1.68 kg m^−2^ h^−1^ and is even faster than the one reported by other systems under 2 sun illumination.

The advent of the steam engine was one of the key developments that led to the first Industrial Revolution. Since then, the use of steam has influenced many aspects of modern life. For instance, thermal steam generation and condensation was one of the dominant technologies for seawater desalination before the introduction of reverse osmosis technologies.^1^, ^2^ Although membrane‐based technologies became the dominant solution to desalination, they are usually energetically demanding with serious environmental impacts arising from cleaning and maintenance.^2^, ^3^ As a result, there is emerging global interest in developing alternative desalination technologies to address these issues.^4^ Solar vapor generation with no electrical input is proving to be a promising and environmentally benign solution, especially in resource limited areas. However, conventional techniques for generating solar vapor typically rely on costly and cumbersome optical concentration systems to enable bulk heating of a liquid, resulting in relatively low efficiencies (e.g., 30–40%) due to heat absorption throughout the entire liquid volume that is not directly translated into vapor production. Recently, various advanced and expensive metallic plasmonic^5^, ^6^, ^7^, ^8^, ^9^, ^10^, ^11^, ^12^, ^13^ and carbon‐based nanomaterials^14^, ^15^, ^16^, ^17^, ^18^, ^19^, ^20^, ^21^, ^22^, ^23^ have been explored for use in solar vapor/steam generation. However, the vaporization efficiencies of these reported structures are still relatively low under 1 sun illumination (e.g., from 48%^10^ to 83%^21^). Very recently, we demonstrated a cost‐effective strategy using thermally isolated carbon‐coated paper (CP) on polystyrene foam with a record high efficiency of ≈88% in the laboratory environment.^24^ To enhance the vapor generation rate, typically the approach is to increase the operational temperature for a given solar illumination. In most literature on solar‐driven vapor generation using advanced nanomaterials (e.g., refs. 12, 13, 16, 19), moderate concentration was usually introduced to enhance the vapor generation rate from a given area of water evaporator. One of the major purposes was to reduce the required area for expensive water evaporators. Due to the limited solar energy (i.e., ≈1 kW m^−2^ under 1 sun illumination) and required energy for liquid‐to‐vapor phase change (e.g., ≈2450 kJ kg^−1^ near ambient temperature), single stage solar vapor generation technologies with no thermal recycling intrinsically require large areas to collect sufficient solar energy for evaporation. Therefore, solar concentration strategy is valid when the evaporator material is more expensive than that of the concentrator or high temperature steam is required (e.g., in conventional solar–thermal multieffect desalination, multistage‐flash desalination, etc.^25^) but not applicable to extremely low cost strategies for portable and distributed applications, such as the CP–foam structure. In addition, in single stage evaporation systems, solar concentration will inevitably increase the thermal loss to the surroundings mainly via conduction, convection, and radiation losses. Therefore, high temperature solar vapor generation using expensive nanomaterials (e.g., with solar concentration^12^, ^13^, ^16^, ^19^) inherently suffers from limits in energy conversion efficiencies.

In this report, we will explore the opposite approach, using solar energy to generate cold vapor below room temperature. This is a breakthrough pathway for efficient solar vapor generation since under illumination at low power densities, the absorbed‐light‐to‐vapor energy conversion efficiency can reach ≈100% when the evaporation temperature is lower than the room temperature. Under this condition, the environment will provide additional energy for vapor generation, resulting in a total vaporization rate that is higher than the upper limit that can be produced using the input solar energy alone. We will provide experimental validation of this cold vapor generation technique with limit‐breaking vaporization rates using an extremely low cost CP–foam system.^24^ Since the cost of the evaporative material is much lower than that of solar concentrator elements, the proposed architecture is more suitable for the development of affordable high performance solar still systems with no solar concentration for personal water purification in resource limited areas.

Loss channels in solar vapor generation systems and the strategy to realize near perfect efficiency: As illustrated in **Figure** **1**A, major loss channels include net radiation, convection, and conduction losses.^12^, ^16^, ^24^, ^26^ Therefore, the power flux consumed by solar‐driven evaporation, *P*_evap_, can be described as^26^

(1) $$P_{\mathrm{evap}}\;=\;P_{\mathrm{light}}\;-\;P_{\mathrm{environment}}\;=\;\alpha C_{\mathrm{opt}}q_{i}\;-\;\varepsilon \sigma (T_{2}^{4}\;-\;T_{1}^{4})\;-\;h\left(T_{2}\;-\;T_{1}\right)\;-\;q_{\mathrm{water}}$$

**Figure 1:**
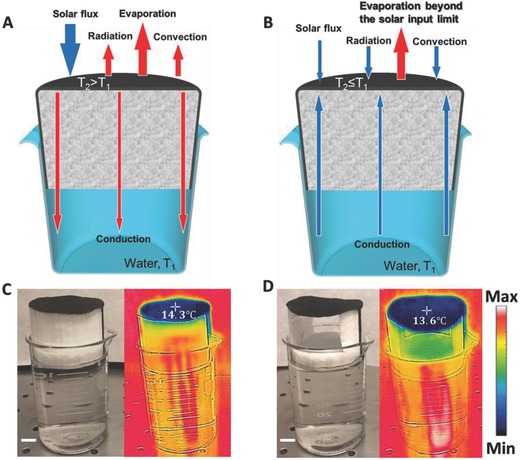
Physical mechanism of vapor generation. A) Energy balance and heat transfer diagram of the CP–foam under strong solar illumination. The surface temperature, *T*_2_, is higher than the room temperature, *T*_1_. B) Energy balance and heat transfer diagram of the CP–foam under dark environment or low intensity illumination. C) A photograph of CP–foam floating on top of water surface and its corresponding thermal image under dark environment. Its surface temperature is below room temperature. D) A photograph of a CP–air–foam structure floating on top of water and its corresponding thermal image under dark environment. Its surface temperature is even lower than the CP–foam structure. Scale bar: 1 cm.

Here, the light input power, *P*_light_ = *αC*_opt_ *q*_i_, α is the optical absorption coefficient, *C*_opt_ the optical concentration, *q*_i_ the normal direct solar irradiation (i.e., 1 kW m^−2^ for 1 sun at AM 1.5). The power flux exchanged with the environment, *P*_environment_ = *εσ*(*T*_2_^4^ − *T*_1_^4^) + *h*(*T*_2_ − *T*_1_) + *q*_water_, ε the optical emission, σ the Stefan–Boltzmann constant (i.e., 5.67 × 10^−8^ W m^−2^ K^−4^)), *T*_2_ the temperature at the surface of the evaporative material, *h* the convection heat transfer coefficient, and *q*_water_ the heat flux to the bulk water. Since the natural evaporation of the bulk water was largely suppressed, its temperature is approximately identical to the environment (see Figure S1 in the Supporting Information). For simplicity, here we use a single *T*_1_ to indicate the temperature of the adjacent environment. This equation describes most major processes (if not all) involved in the evaporation process, i.e., the absorption of light, *αC*_opt_ *q*_i_, the net radiative loss to the surroundings, *εσ*(*T*_2_^4^ − *T*_1_^4^), the convective loss to the ambient, *h*(*T*_2_ − *T*_1_), and the radiative and conductive loss to the bulk water, *q*_water_. By manipulating the energy distribution among these channels, unique solar vapor generation mechanisms can be realized. For instance, in a recently reported strategy, a selective absorber and a bubble wrap cover were introduced to decrease the infrared thermal radiation (ε) and the convective loss (*h*) to the surroundings, respectively, to produce 100 °C steam under 1 sun illumination.^26^ However, for high temperature (*T*_2_ > *T*_1_) solar vapor generation systems, these losses can only be reduced but not eliminated completely (i.e., *P*_evap_ < *P*_light_). Therefore, an important question is what happens when *T*_2_ ≤ *T*_1_? This condition will be the major focus of our work.

As illustrated in Figure 1B, in a steady case (with a stable surface temperature *T*_2_ ≤ *T*_1_), Equation (1) will be modified slightly as

(2) $$P_{\mathrm{evap}}\;\;=\;\;P_{\mathrm{light}}\;\;+\;\;P_{\mathrm{environment}}\;\;=\;\;\alpha C_{\mathrm{opt}}q_{i}\;\;+\;\;\varepsilon \sigma (T_{1}^{4}\;-\;T_{2}^{4})\;+\;h\left(T_{1}\;-\;T_{2}\right)\;\;+\;\;q_{\mathrm{water}}$$

In this case, the system will actually take energy from the environment (i.e., *P*_evap_ > *P*_light_) and the absorbed solar energy will only be consumed in the liquid‐to‐vapor phase transition, corresponding to near perfect solar energy conversion! Next, we employ our recently reported thermally isolated CP on foam^24^ as a low cost testbed to analyze the energy balance and heat transfers under both dark and illuminated conditions (see Section S1 in the Supporting Information for details of material and sample preparation).

Water evaporation is a natural process which occurs under any conditions regardless of solar illumination.^27^ According to the classical kinetic theory of gases,^28^ water molecules near the air–water interface that move toward the interface with sufficient kinetic energy to overcome liquid‐phase intermolecular forces can evaporate. The evaporation rate is dependent on many factors, including heat (or temperature), atmospheric pressure (including the humidity), air movement (including convection), and water salinity. One can refer to Section S2, Figure S2, and Tables S1 and S2 (Supporting Information) for experimental characterization of vapor generation rates of CP samples under dark environment with well‐controlled temperature and humidity using a commercial glove box. For simplicity, the effect of salinity was not considered in this work.

As shown in Figure 1C, a ≈20 cm^2^ CP was attached to a foam substrate floating on top of water. Two strips of CP in contact with the underlying water transport the water to the upper surface for evaporation by the capillary forces. Its surface thermal distribution was then characterized using a portable thermal imager (FLIR ONE) once the temperature was stable. One can see that the surface temperature of the CP is ≈14.3 ± 0.2 °C (*T*_2_), which is lower than that of the room temperature (i.e., *T*_1_ = 22.3–23.3 °C). According to our characterization under the laboratory environment (with the humidity of 16–25% in winter time at Buffalo), the average evaporation rate in the dark environment is 0.275 kg m^−2^ h^−1^. Due to natural evaporation, this process will consume 6.78 × 10^5^ J m^−2^ h^−1^ energy from the environment (considering the enthalpy of vaporization at 14.3 °C^29^). Therefore, the energy balance and heat transfer diagram under dark environment (or low intensity illumination condition) is different from that in previously reported solar heating situation. As shown in Figure 1B, the heat transfer is actually from the environment to the CP surface due to the lower temperature of the sample. According to Equation (2), the convective input power, *P*_con_ = *h*(*T*_1_ − *T*_2_), is ≈2.88 × 10^5^ J m^−2^ h^−1^ (*h* is assumed to be 10 W m^−2^ K^−1^ 26) under dark conditions. This heat transfer direction will be valid as long as the CP surface temperature is lower than the surrounding temperature. In addition, the system has no net radiation loss when *T*_2_ ≤ *T*_1_. Instead, according to the equation *P*_rad_ = *εσ*(*T*_1_^4^ − *T*_2_^4^) (ε is 0.969 for the CP, see Figure S3 in the Supporting Information), the radiative input power can be calculated to be 1.56 × 10^5^ J m^−2^ h^−1^. The remaining input is contributed by *q*_water_ from the paper dipped in the water and the foam substrate. Therefore, the CP foam system actually takes energy from the environment rather than loses it. From this standpoint, the ideal material/structure for solar vapor generation should have a higher evaporation rate under dark conditions in order to achieve a lower equilibrium temperature. As a result of this insight, we removed the foam under the CP to introduce an air gap (CP–air–foam), the evaporation rate was then enhanced to 0.34 kg m^−2^ h^−1^, resulting in a lower temperature of ≈13.6 °C at the CP surface as shown in Figure 1D. To examine how this arrangement influences our solar vapor generation, we introduce light illumination to accelerate the vapor generation.

In this experiment (see Section S1 in the Supporting Information for details), we employed a solar simulator (Newport) to illuminate the CP samples (**Figure** **2**A and Figure S4 (Supporting Information)). The light beam is filtered by an optical diffuser (see Figure S3 in the Supporting Information) to get a more uniform beam spot with the power density of ≈0.6 kW m^−2^ (i.e., equivalent to the power of 0.6 sun at AM1.5). However, the temperature distribution is not uniform due to the divergence of the beam from the solar simulator. One can see that the surface temperature of the central part of the CP–foam sample (upper panel in Figure 2B) increased up to 35.3 °C, while the CP–air–foam (lower panel in Figure 2B) surface temperature increased up to 29.7 °C. They are both higher than the room temperature. Therefore, the loss channels highlighted in Figure 1A will result in lower solar energy conversion efficiency in these areas. One can see from Figure 2C that these measured average evaporation rates (i.e., 0.68 kg m^−2^ h^−1^ (purple spheres) and 0.80 kg m^−2^ h^−1^ (orange spheres)) are both below the upper limit that can be produced by the input solar energy (i.e., ≈0.90 kg m^−2^ h^−1^, the solid curve). It should be noted that the CP–air–foam sample realized a better vapor generation rate under the same illumination, confirmed by its lower surface temperature.

**Figure 2:**
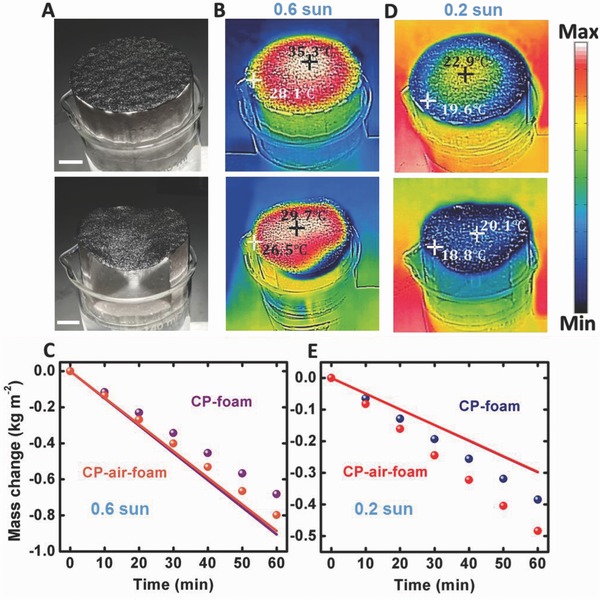
Vapor generation under low density light illumination. A) Photographs of a CP–foam (upper panel) and a CP–air–foam (lower panel) under 0.6 sun illumination. B) Thermal images of the CP–foam (upper panel) and the CP–air–foam (lower panel) under 0.6 sun illumination. C) Comparison of measured water weight change versus time of CP–foam (purple spheres) and CP–air–foam (orange spheres). The upper limit that can be produced by 0.6 sun input solar energy is plotted by the solid curve. D) Thermal images of the CP–foam (upper panel) and the CP–air–foam (lower panel) under 0.2 sun illumination. E) Comparison of measured water weight change versus time of CP–foam (blue spheres) and CP–air–foam (red spheres). The upper limit that can be produced by 0.2 sun input solar energy is plotted by the solid curve. The two solid curves are close to each other due to the similar surface temperatures shown in (D). Scale bar: 1 cm.

To minimize these loss channels, we reduced the incident power to ≈0.2 kW m^−2^. As shown by the upper panel in Figure 2D, the central area temperature of the CP–foam structure was reduced to 22.9 °C. Other areas on this sample are all below room temperature. In addition, the highest temperature of the CP–air–foam structure was 20.1 °C (lower panel in Figure 2D), all below room temperature. Under this situation (i.e., Figure 1B), we obtained the total vapor generation rate of 0.39 kg m^−2^ h^−1^ for the CP–foam sample and 0.48 kg m^−2^ h^−1^ for the CP–air–foam sample, respectively, as shown by spheres in Figure 2E. Remarkably, they are all beyond the theoretical upper limit of the vapor generation rate that can be produced by the input solar energy (i.e., ≈0.30 kg m^−2^ h^−1^, the solid curve in Figure 2E). It should be noted that we did not subtract the dark evaporation “background.” Next, we will explain why this “background” should not be removed by analyzing the thermodynamic process under different operation conditions.

In previously reported solar vapor generation literature, the dark evaporation was usually considered as a background which was subtracted from the total vapor generation to obtain the net solar‐induced vapor generation and calculated the solar energy conversion efficiency. However, by simply comparing Figure 1A, and Figure 1B, one can see that the energy balance and heat flow direction under dark conditions are different from those under illuminated conditions. Therefore, the dark evaporation should not be a part of the evaporation under solar illumination. Admittedly, this interpretation adjustment is not a big factor to previously reported literature since the dark evaporation rate is usually negligible compared with those rates obtained under solar concentration. But, it is more important in our low intensity illuminated condition. Importantly, by analyzing the transient thermal and mass change conditions by changing the illumination intensity, the thermal dynamic process can be revealed.

As shown in **Figure** **3**A, we recorded the mass change of the CP–foam system when the light (0.6 sun) was turned off at the time of ≈162 s (indicated by the arrow). One can see that the solar‐driven evaporation rate (the red dashed line) changed to the dark rate (the purple dashed line) after ≈2 min (see Movie S1 in the Supporting Information for the entire thermal process). During this 2 min period, the evaporation rate is always higher than the dark rate due to the solar–thermal energy stored in the evaporator system. However, the widely used definition of the solar energy conversion efficiency (e.g., refs. 9, 10, 11, 12, 13, 16, 18, 19, 20, 21, 26, 30) is obviously not applicable in this transient process since there is no solar input. Therefore, the physical picture of the solar‐driven evaporation should be revisited.

**Figure 3:**
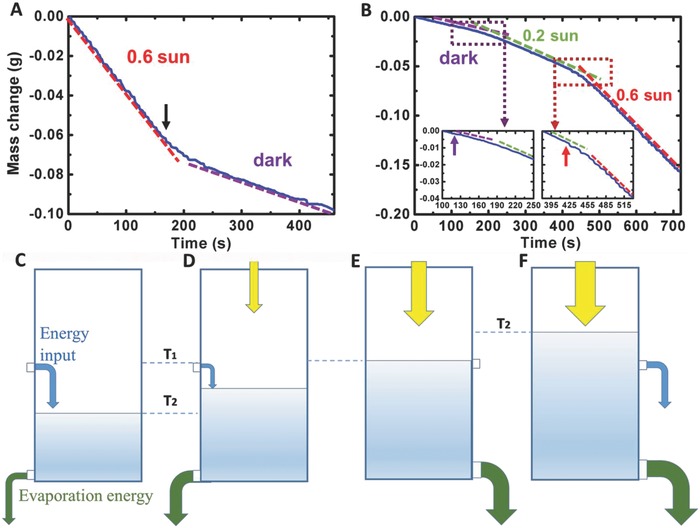
Physical interpretation of the thermal dynamic energy balance of solar vapor generation systems. A) Continuously measured mass change from 0.6 sun illumination to dark conditions. B) Continuously measured mass change from dark to 0.2 sun and then to 0.6 sun conditions. Dashed lines indicate the fitted mass change rates. Insets: Zoom‐in data from dark to 0.2 sun (left panel) and from 0.2 sun to 0.6 sun conditions (right panel). C) Energy flow diagram under dark conditions: the input energy from the environment is in balance with the evaporation energy. D) Energy flow diagram of a below‐room‐temperature system with a weak light input: the output evaporation energy is the sum of the light input and the environment input. E) Energy flow diagram of a room‐temperature system: the output evaporation energy is in balance with the surrounding and light input. F) Energy flow diagram of a hot system: the input solar energy is the sum of the evaporation energy and the loss to the environment.

To describe the transient thermodynamic process, the energy stored or released by the evaporator system can be investigated using the time‐dependent power flux equation.

(3) $$\begin{array}{l} P_{\mathrm{evap}}\left(t\right)\;=\;P_{\mathrm{light}}\left(t\right)\;+\;P_{\mathrm{environment}}\left(t\right)\\ \;\;\;\;\;\;\;\;\;\;\;=\alpha C_{\mathrm{opt}}q_{i}\left(t\right)\;-\;\varepsilon \sigma \;[T_{2}^{4}\left(t\right)\;-\;T_{1}^{4}]\;-\;h[T_{2}\left(t\right)\;-\;T_{1}]\;-\;q_{\mathrm{water}}\left(t\right) \end{array}$$

When the solar input is turned on or turned off, the system will store or release energy depending on the thermal capacity of the system. To interpret this intriguing problem, we recorded a continuous weight change of the CP system under different illumination conditions. As shown in Figure 3B, the solar simulator was turned on at the time of 120 s (purple arrow) with the intensity of 0.2 sun and was then tuned to 0.6 sun at the time of 420 s (red arrow). Their energy balance will be analyzed using a “water container” model, as illustrated in Figure 3C–F.

Under dark conditions (Figure 3C), the energy consumed by natural evaporation is in balance with the input energy from convection, conduction, radiation, and others (if any). The system temperature *T*_2_ is lower than the room temperature *T*_1_ (e.g., Figure 1C,D). The dark evaporation of the system is dependent on environmental conditions (e.g., the temperature, humidity, pressure, system architecture, etc.), as indicated by the purple dashed line in Figure 3B. In this situation, there is one input (i.e., the environment) and one output (i.e., the dark evaporation).

When we introduce a relatively weak solar energy input (e.g., 0.2 sun at purple arrow in Figure 3B), the system temperature *T*_2_(*t*) will increase (Figure 3D). Part of the absorbed solar energy will result in this temperature increase and the rest is consumed in the liquid–vapor transition (see the bending curve from the purple dashed line to the green dashed line in the left inset in Figure 3B). During this transient process, the system will store extra energy from the solar input. For instance, at the time starting from ≈120 s, the surface temperature of the system increased from 14.3 to ≈14.8 °C in ≈10 s (the 10 s period was selected due to the accuracy in our mass change measurement). During this 10 s period, the average temperature is ≈14.6 °C and the evaporation rate is ≈0.26 kg m^−2^ h^−1^. Since the average temperature is lower than the ambient, the system still took energy from the environment. Therefore, in this transient process, there are two inputs (i.e., the solar input and the environmental input) and two outputs (i.e., water evaporation and temperature increase of the system). Using measured average data, the energy distribution ratio in the input and output channels can then be estimated (see calculation details in Section S3.2 in the Supporting Information): for the input channel, the solar input contribution is ≈54.7% and the environmental contribution is ≈45.3%; for the output channel, only ≈51.8% of the total input energy within this 10 s transient period was consumed by the evaporation. The rest ≈48.2% was stored in the system and resulted in the observed temperature increase. Importantly, this energy distribution ratio continues to change during this transient process. For example, at the next 10 s period (e.g., 130–140 s), the surface temperature of the system increased from ≈14.8 to ≈15.6 °C and the average evaporation rate is ≈0.31 kg m^−2^ h^−1^, respectively. Due to the temperature rise in this 10 s period (i.e., smaller temperature difference between the surface and the ambient temperature), the solar input contribution increased to ≈56.8% and the environmental contribution decreased to ≈43.2%. For the output channel, ≈64.2% of the total input energy within this 10 s period was consumed in the evaporation, while the rest ≈35.8% was stored in the system and resulted in the further increased temperature.

This heat storage process continued until the surface temperature stabilized at ≈22.9 °C (i.e., the upper panel in Figure 2D). Then, the system reached a new steady state with an evaporation rate of ≈0.39 kg m^−2^ h^−1^ (Figure 2E). After that, the total input energy can only go to the evaporation output channel (i.e., a two‐input and one‐output system). In this steady state, the total input energy was contributed by the solar input and the environment by ≈61.5% and ≈38.5%, respectively. One can see that under low‐intensity illumination, the environment contribution plays an important role in the evaporation. As long as the system's steady‐state temperature is still lower than the room temperature *T*_1_ (e.g., Figure 2D), the absorbed solar energy will be solely transferred to the liquid‐vapor transition (because there is no thermal loss to the environment). Under this steady‐state condition, the total evaporation is higher than that can be produced by the input solar energy due to the extra input from the environment (e.g., Figure 2E).

When the system temperature increases up to the room temperature with stronger solar input (Figure 3E), the input energy channel from the environment will be closed. Ultimately, the output energy consumed by the evaporation is in balance with the input solar energy under the new steady state with perfect conversion efficiency. If the input solar energy is increased further, as illustrated in Figure 3F (e.g., 0.6 sun in Figure 3B), the system temperature *T*_2_(*t*) is higher than *T*_1_ (e.g., Figure 2B). During this transition process, the system will store more energy from the solar input (i.e., the bending curve from the green dashed line to the red dashed line in the right inset in Figure 3B), which will result in loss to the environment. In this case, the evaporation energy is always smaller than the input solar energy. Therefore, the absorbed solar energy conversion efficiency is smaller than 100% and the obtained vapor generation rate cannot surpass the theoretical upper limit (e.g., Figure 2C). More details on energy distribution estimation for 0.6 sun illumination are listed in Section S3.1 (Supporting Information).

Although Figure 2E demonstrated the limit breaking solar‐driven vapor generation, the total vapor generation rate is still relatively low due to the weak solar illumination. The most important question is how to realize this below‐room‐temperature strategy under a practical 1 sun illumination. A straightforward method is to increase the actual surface area within a given projection area, as illustrated in **Figure** **4**A. To demonstrate this strategy, we fabricated a set of triangle structures with different apex angles (θ) and compared their surface temperature distributions with a flat sample. As shown in Figure 4B, the highest temperature on the flat CP sample is 42.6 °C. The measured mass change and the theoretical upper limit data (normalized to the projection area) are plotted in Figure 4C. Since the surface temperature of the flat CP sample is higher than the room temperature, corresponding to the lossy system in Figure 3F, the measured vapor generation rate (≈1.21 kg m^−2^ h^−1^, see purple spheres) is lower than that of the theoretical limit (≈1.58 kg m^−2^ h^−1^, the purple curve).

**Figure 4:**
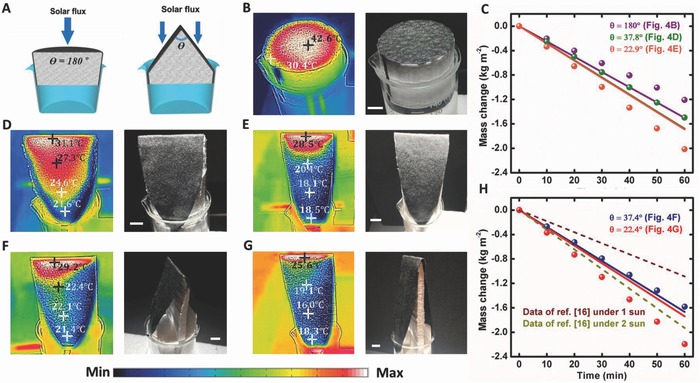
Increased surface area under 1 sun illumination. A) Schematic diagram to reduce the light density by introducing larger surface area structures. B,D,E) The thermal distribution images and corresponding photographs of (B) a flat CP–foam, (D) a triangle structure with θ of 37.8°, (E) a triangle structure with θ of 22.9°. C) Comparison of measured water weight change versus time of these three CP–foam samples (spheres). The upper limits that can be produced by 1 sun input solar energy are plotted by solid curves. F,G) The thermal distribution images and corresponding photographs of CP–air–foam structures with (F) θ = 37.4° and (G) θ = 22.4°. H) Comparison of measured water weight change versus time of these two CP–air–foam samples (spheres). The upper limits that can be produced by 1 sun input solar energy are plotted by solid curves. The weight change under 1 and 2 sun illumination reported by Ghasemi et al.^16^ are plotted by dashed curves. Scale bar: 1 cm.

When we employed the same light to illuminate the triangle samples with larger surface areas (Figure 4D,E), the temperature decreases significantly compared with the flat sample shown in Figure 4B. It should be noted that all samples were characterized after 30 min stabilization under 1 sun illumination. As shown by Movie S3 (Supporting Information), the stabilization time is ≈2 min. Here, we indicate four temperature points at different areas along the side walls. One can see that a major area of the sample in Figure 4D (θ = 39°) is still higher than the room temperature. As a result, we observed a total evaporation rate of ≈1.50 kg m^−2^ h^−1^, which is ≈88.9% of the input solar energy (see green spheres and the green curve in Figure 4C). This efficiency is improved compared with the flat CP sample in Figure 4B. More intriguingly, to realize the 0.2 sun illumination power density (Figure 2D) under 1 sun condition, the surface area of the CP need to be enhanced by 5 times. Therefore, the apex angle (θ) should be ≈23° (i.e., sin(θ/2) = 1/5). In this case, the vapor generation rate is expected to be enhance by ≈5× compared with the one observed in Figure 2D. As shown in Figure 4E, we fabricated a sample with the θ of 22.4°. One can see that the surface temperature was decreased further with major areas below room temperature. In this case, we observed a total vapor generation rate of ≈2.02 kg m^−2^ h^−1^ (orange spheres in Figure 4C), which is higher than the theoretical upper limit (≈1.65 kg m^−2^ h^−1^, see the orange curve in Figure 4C). Ultimately, we removed the foam under these two triangle samples to get CP–air triangle samples to further enhance the convection contribution from the surroundings and accelerate the evaporation rate. As shown by Figures 4F,G, the surface temperatures can be reduced further under the same illumination conditions, indicating the improved vapor generation rates. As shown in Figure 4H, we obtained total vapor generation rates of 1.58 kg m^−2^ h^−1^ for the sample in Figure 4F and 2.20 kg m^−2^ h^−1^ for the sample in Figure 4G, respectively. In particular, the best result of 2.20 kg m^−2^ h^−1^) is ≈4.6× of the one observed in Figure 2D (i.e., 0.48 kg m^−2^ h^−1^), even faster than those reported by other systems under 1–2 sun illumination (e.g., ≈1.09 kg m^−2^ h^−1^ under 1 sun and ≈1.93 kg m^−2^ h^−1^ under 2 sun reported by Ghasemi et al.,^16^ see dashed lines in Figure 4H). By comparing the total vapor generation rate and the theoretical upper limit in Figure 4C,H, one can see that the structures with larger surface areas in Figure 4E,G (θ = 23°–24°) took ≈16.0% and ≈20.7% energy, respectively, from the environment for vapor generation. This encouraging result indicates the potential to realize ultraefficient and high performance solar stills based on extremely low cost materials. By reducing the apex angle θ, the surface area of the triangle sample can be increased further. The practical limit of the apex angle (≈8.4°) is determined by the water transportation limit of the paper strip (see Figure S5 in the Supporting Information).

In addition, we recognized that the temperature at the top of the triangle sample is still higher than the room temperature. These “hot” regions are mainly introduced by the non‐uniform incident light: in our experiment, the solar intensity at different heights is slightly different due to the divergence of the beam from the solar simulator. Therefore, the maximum temperatures are above room temperature at some regions. To obtain a more accurate understanding of the energy conversion process, we used the stronger solar intensity at the top position as the input intensity to calculate the solar‐driven vapor generation limit (see Section S4 and Table S3 in the Supporting Information). Therefore, the limit‐breaking experimental result is unambiguous. On the other hand, it indicates that the experimental evaporation rate observed in Figure 4 can be optimized further because the system still loses some energy to the environment in the hot regions. More details under uniform solar illumination in an outdoor environment are discussed in Figure S6 (Supporting Information).

In conclusion, for practical outdoor solar still applications, stable and continuous solar illumination is not achievable in most areas of this planet due to varying weather conditions and sun position. Even with inexpensive moderate solar concentrators (e.g., ref. 16), a stable incident power higher than AM 1.5 solar light still cannot be guaranteed. Additionally, since most solar stills are covered by glass or other similar collection material, condensation can lead to optical scattering and a decrease in the incident solar power. Therefore, vapor generation under 1 sun illumination condition is important, despite being neglected in most previously reported work. As a result, this report explored an alternative approach to solar vapor generation using cold vapor below room temperature, and revealed a near unity conversion efficiency of absorbed solar energy. Due to the energy contribution from the surroundings, the measured total vapor generation is higher than the upper limit that can be produced by a given incident solar energy. Importantly, this breakthrough technique was realized using an extremely low cost CP–foam system under 1 sun illumination, with no need for advanced and expensive nanomaterials. In addition, we also revealed the key features for optically absorbing and evaporative materials for future solar still systems,^31^ i.e., under a given environment, a stronger natural evaporation capability will result in a lower surface temperature. This indicates a novel research direction to explore and develop different evaporative materials, which may have applications in solar still technology, evaporative cooling,^32^ solar‐evaporated mining applications,^33^ evaporation‐driven generators,^34^, ^35^ and recently reported water‐evaporation‐induced electricity.^36^ In addition, this cold vapor generation will raise a new scientific question on moisture condensation for water collection.^37^ It is challenging to release the thermal energy from these below room‐temperature vapor with no electricity. Thus, we anticipate that integration of this evaporative regime with the recently emerging day‐time radiative cooling systems^38^, ^39^, ^40^, ^41^ will initiate a revolution in thermodynamic technologies.

## Conflict of Interest

The authors declare no conflict of interest.

## Supporting information

Supplementary

Supplementary

Supplementary

Supplementary
