# Supplementary material for: Cold Vapor Generation beyond the Input Solar Energy Limit
Source: Adv Sci (Weinh). 2018 May 3;5(8):1800222. doi: 10.1002/advs.201800222 (PMC6096986; doi:10.1002/advs.201800222)
Supplement: Supplementary file 1 — Supplementary [file ADVS-5-1800222-s002.pdf]

Copyright WILEY-VCH Verlag GmbH & Co. KGaA, 69469 Weinheim, Germany, 2018.

ADVANCED SCIENCE
Open Access

Supporting Information

for *Adv. Sci.,* DOI: 10.1002/advs.201800222

Cold Vapor Generation beyond the Input Solar Energy Limit

*Haomin Song, Youhai Liu, Zhejun Liu, Matthew H. Singer, Chenyu Li, Alec R. Cheney, Dengxin Ji, Lyu Zhou, Nan Zhang, Xie Zeng, Zongmin Bei, Zongfu Yu, Suhua Jiang, and Qiaoqiang Gan**

Copyright WILEY-VCH Verlag GmbH & Co. KGaA, 69469 Weinheim, Germany, 2018.

Supporting Information

Cold vapor generation beyond the input solar energy limit

Haomin Song,‡[a] Youhai Liu,‡[a] Zhejun Liu,[b] Matthew H. Singer,[a] Chenyu Li,[a] Alec R. Cheney,[a] Dengxin Ji,[a] Lyu Zhou,[a] Nan Zhang,[a] Xie Zeng,[a] Zongmin Bei,[a] Zongfu Yu,[c] Suhua Jiang,[b] Qiaoqiang Gan*[a]

[a] *Department of Electrical Engineering, The State University of New York at Buffalo, Buffalo, NY 14260, USA. Email: qqgan@buffalo.edu*

[b] *Material Science Department, Fudan University, Shanghai 200433, China*

[c] *Department of Electrical and Computer Engineering, University of Wisconsin, Madison, Wisconsin 53705, USA*

‡ These two authors contributed equally to this work.

1. Materials and Methods

Materials: The materials used to fabricate the carbon-coated paper (CP) are commercially available: paper (Texwipe™ TX609) and carbon powder (Sid Richardson Carbon & Energy Co.).

Sample fabrication: 2 g carbon powder was dispersed into 400 mL water. 8 mL acetic acid was added to make carbon powder easier to attach to fibers.[1] The solution was mixed in a 1000 ml beaker and blended well using an ultrasonic cleaner (Branson Ultrasonics Bransonic™ B200) for 5 minutes. Subsequently, the prepared white paper was put into the mixed solution to vibrate and stir for 3 minutes so that carbon powders can dye the paper uniformly. After that, the CP was dried at 80 ºC on a heating stage. This procedure was repeated three to four times to realize the ideally dark color.

Sample characterization: We characterized the absorption spectrum using an integration sphere spectroscopy (Thorlabs IS200-4 integrated with Ocean Optics USB2000+, Ocean Optics Jaz, and Avantes AvaSpec-NIR256-1.7TEC for ultraviolet, visible and infrared wavelength range, respectively). By weighting the optical absorption spectrum of CP (see the blue curve in fig. S3 in the ESI†) with the AM 1.5 solar irradiance (see the red spectrum), the optical absorption of the CP sample is ~96.9%.

Solar vapor generation: To measure the water evaporation rate, a 150 mL beaker with an inner diameter of 5 cm filled with ~140 g water was placed under an intensity-tunable solar simulator (Newport 69920), as shown in fig. S4. Three diffusers (10 inch × 8 inch × 0.050 inch polystyrene sheet, Plaskolite) were used to generate a uniform light distribution. As shown by the orange curve in fig. S3, their overall transmission spectrum is almost wavelength-independent. Therefore, the diffusers did not change the spectral feature of the incident light. The solar light intensity was

measured using a power meter (PM100D, Thorlabs Inc.) equipped with a thermal sensor (S305C, Thorlabs Inc.) at the same height of the CP. The CP was first illuminated for approximately 30 minutes for stabilization. Then the evaporation weight change was measured by an electronic scale (U.S. Solid, with the resolution of 1 mg) every 10 minutes. The surface temperature of CP was characterized using a portable thermal imager (FLIR ONE®). The spatial resolution in our characterization is 0.1~0.2 mm (which is dependent on the distance between the camera and the object). The measured temperature resolution claimed in the manual from the manufacturer is 0.1 °C and the accuracy is 3-5% to the difference between ambient and scene temperature. To calibrate the temperature, a piece of white paper without illumination was adopted as a reference for room temperature in the same thermal imaging. Its temperature shown in the thermal distribution image was calibrated by a thermometer (GoerTek). In this case, the error in the temperature characterization due to distance from the sample to the thermal imager can be minimized. In addition, we performed extra characterization to compare the temperature measured in thermal imaging with the one characterized by the thermal couple probe. As shown in **fig. S7**, the accuracy in the thermal image characterized in our laboratory environment is reasonably good. All thermal images shown in the main text were characterized at a stable state. Details of surface temperature change of a CP sample over short time (5 minutes) and long time (3 hours) are shown in **movie S2** and **fig. S8**, respectively.

2. Dark evaporation in controlled environment

It is known that the evaporation rate is strongly related to the environment conditions. To obtain the direct data in our laboratory environment, we characterized the dark evaporation rate of bare water surface in a glove box with controlled relative humidity and temperature (ETS Model 5501-11, electro-tech system, Inc., fig. S2). In this experiment, we performed two sets of measurement by fixing the relative humidity and temperature inside the box, respectively. Each condition was stabilized for 1 hour before the characterization. The higher relative humidity and/or lower environment temperature will result in lower dark evaporation rates (table S1). Besides, the higher relative humidity will lead to higher surface temperature (table S2). We also compared the surface temperature of the bare water and the CP. Under the same temperature and humidity, the evaporation rate of CP sample is faster than that of the bare water surface. Therefore, the surface temperature of CP is lower than the bare water (table S2).

3. Energy distribution calculation

According to the detailed characterization of the evaporation under different solar illumination conditions shown in Fig. 3, transient processes were emphasized, which were largely ignored in previous literature. During these steady and transient states, the energy distribution can be estimated by considering experimental results using the time-dependent energy balance equation (3).

3.1 Steady states

Under 0.2 sun illumination, since the surface temperature is lower than the ambient, we have two inputs (i.e., solar input and the environmental input) and one output (i.e., the water evaporation). According to our characterization of the CP-air-foam sample shown in Fig. 2E (under 0.2 sun), the measured steady-state evaporation rate is 0.2972 kg m^{-2} h^{-1} (red spheres) and the theoretical upper limit of the vapor at 22.9 °C is ~0.4836 kg m^{-2} h^{-1} (see Section 4 in the Supporting Information). Consequently, the total input energy was contributed by the solar input and the environment by ~61.5% (i.e., 0.2972/0.4836 = 61.5%) and ~38.5%, respectively.

Under 0.6 sun illumination, since the surface temperature is higher than the ambient, we have one input (i.e., solar input) and two outputs (i.e., the water evaporation and the loss to the

environment). According to our characterization of the CP-foam sample shown in Fig. 2C (under 0.6 sun), the measured steady-state evaporation rate is 0.6818 kg m^{-2} h^{-1} (purple spheres) and the theoretical upper limit of the vapor at 29.7 °C is ~0.9060 kg m^{-2} h^{-1}. In this case, the total loss to the environment under the steady state is ~24.7% [i.e., (0.9060-0.6818)/0.9060 = 24.7%].

3.2 Transient processes

The energy balance situation in transient processes is more complicated than those at steady states due to the additional thermal energy change of the system.

During the transient process, the time-dependent surface temperature and the evaporation rate of the system were continuously measured. Using the averaged surface temperature and ambient temperature within a given period (i.e., t = 10 s in our case), one can estimate the power input from solar illumination and environment using equation (3). Therefore, the input contribution ratio of solar illumination (i.e., absorption) and environment (i.e., radiation, conduction and convection) can be calculated. Using the averaged evaporation rate and the enthalpy of vaporization at the averaged surface temperature, one can estimate the energy output via evaporation.

For instance, at the time starting from ~120 s in Fig. 3B, the surface temperature of the system increased from 14.3 °C to ~14.8 °C in ~10 s under 0.2 sun illumination (i.e., 200 W m^{-2}). During this 10 s period, the average temperature is ~14.6 °C and the evaporation rate, m, is ~0.26 kg m^{-2} h^{-1}. Since the average temperature is lower than the ambient, the system still took energy from the environment. Therefore, in this transient process, there are two inputs (i.e., the solar input and the environmental input) and two outputs (i.e., water evaporation and temperature increase of the system). The solar input energy is 1.88×10^3 J m^{-2} (i.e., $\alpha C_{opt}q_i t$ = $0.969\times200\times10/3600$ J m^{-2}). The radiation input is 4.22×10^2 J m^{-2} (i.e., $\varepsilon\sigma(T_1^4- T_2^4)t$ = $0.969\times5.67\times10^{-8}\times[(22.3+273)^4-(14.6+273)^4]\times10$ J m^{-2}). The convection input is 7.75×10^2 J m^{-2} [i.e., $h(T_1 - T_2)t$ = $10\times(22.3-14.6)\times10$ J m^{-2}]. The input of bulk water is 3.60×10^2 J m^{-2} [i.e., $h_{water}(T_1 - T_2)t$ = $4.64\times(22.3-14.6)\times10$ J m^{-2}].

Here we assume that the main heat transfer channel is conduction and convection. The effective heat transfer coefficient, h_{water} = 4.64 W m^{-2} K^{-1}, is obtained by analyzing the steady state of 0.2 sun illumination, and using the equation: $h_{water} = [P_{evaporation} - \alpha C_{opt}q_i + \varepsilon\sigma(T_1^4 - T_2^4) + h(T_1 - T_2)]/(T_1 - T_2)$, where $P_{evaporation}$ is the power consumed by evaporation. Consequently, the environment input (i.e., radiation, conduction and convection) is 1.56×10^3 J m^{-2} [i.e., $(4.22+7.75+3.60)\times10^2$ J m^{-2}]. Therefore, for the input channel, the solar input contribution is ~54.7% [i.e., 1.88/(1.88+1.56) = 54.7%] and the environmental contribution is ~45.3%. The enthalpy of evaporation, h_{vap}, is 2465.491 J g^{-1} at 14.6 °C. Therefore, the output energy consumed by evaporation is 1.78×10^3 J m^{-2} [i.e., $mh_{water}t$ = $0.26\times2465.491\times1000\times10/3600$ J m^{-2}]. The system stores an energy of 1.65×10^3 J m^{-2} (i.e., $[(1.88+1.56)\times10^3-1.78\times10^3]$ J m^{-2}). Therefore, for the output channel, only ~51.8% of the total input energy within this 10 s transient period was consumed by the evaporation [i.e., 1.78/(1.78+1.65) = 51.8%]. The rest ~48.2% was stored in the system and resulted in the observed temperature increase.

4. The upper limit of the vapor that can be generated by the input solar energy

In the main text, we intended to demonstrate a limit-breaking solar vapor generation rate beyond the input solar energy limit. Therefore, the estimation of the theoretical upper limit is important as will be explained in details below.

In this calculation, we assumed that the solar energy was transferred solely to the liquid-vapor transition without any other losses. Therefore, the obtained solar vapor generation rate is equal to the solar intensity (J m^{-2} h^{-1}) divided by the enthalpy of evaporation (J kg^{-1}).

The solar intensity was measured by placing the aforementioned S305C thermal sensor perpendicular to the light beam. For triangle structures shown in Fig. 4, the solar intensity at different height is slightly different due to the divergence of the beam. It should be noted that in our calculation of the vapor generation rate, we actually overestimated the input solar energy: i.e., we assumed that the CP was illuminated by the solar light of the highest intensity and the optical absorption is 100%. Therefore, the limit-breaking experiment result is unambiguous.

For instance, in the left panel of Fig. 4G, we employed the strongest illumination at the top of the triangle sample, 1.181 sun as the solar intensity (i.e., 1.181 kW m^{-2} = 4.2516×10^6 J m^{-2} h^{-1}).

The enthalpy of evaporation is temperature dependent. Therefore, it is important to analyze the temperature distribution on the CP surface, which is non-uniform (Fig. 2 and Fig. 4). The energy flow condition varies on the same CP sample due to the non-uniform temperature distribution. Since the enthalpy of evaporation is smaller at higher temperature,[2] we selected the enthalpy of evaporation corresponding to the highest temperature on the CP surface to calculate the theoretical upper limit. For example, in the left panel of Fig. 4G, we adopted the enthalpy of evaporation of 2444.2 J g^{-1} (i.e., 2.4442×10^6 J kg^{-1}) at 25.6°C (i.e., the highest temperature on the CP surface).

Therefore, under the 1.181 sun solar illumination, the theoretical upper limit of the vapor generation rate is 1.739 kg m^{-2} h^{-1}. All values used in the calculation are listed in table S3.

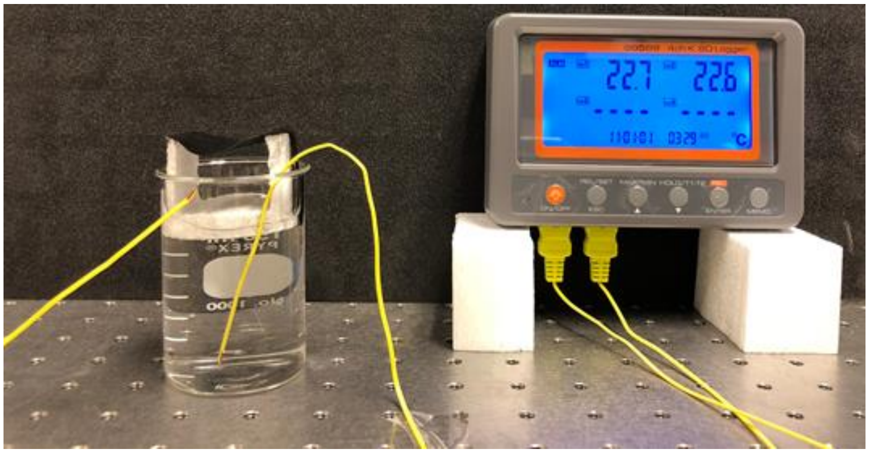

Fig. S1. Characterization of the environmental temperature. One probe is in the bulk water under the CP foam structure, and the other probe is outside the beaker. Since the natural evaporation of the bulk water was largely suppressed, its temperature is almost identical to the environment. For simplicity, we used a single T_1 in eq. (1) to indicate the temperature of the adjacent environment.

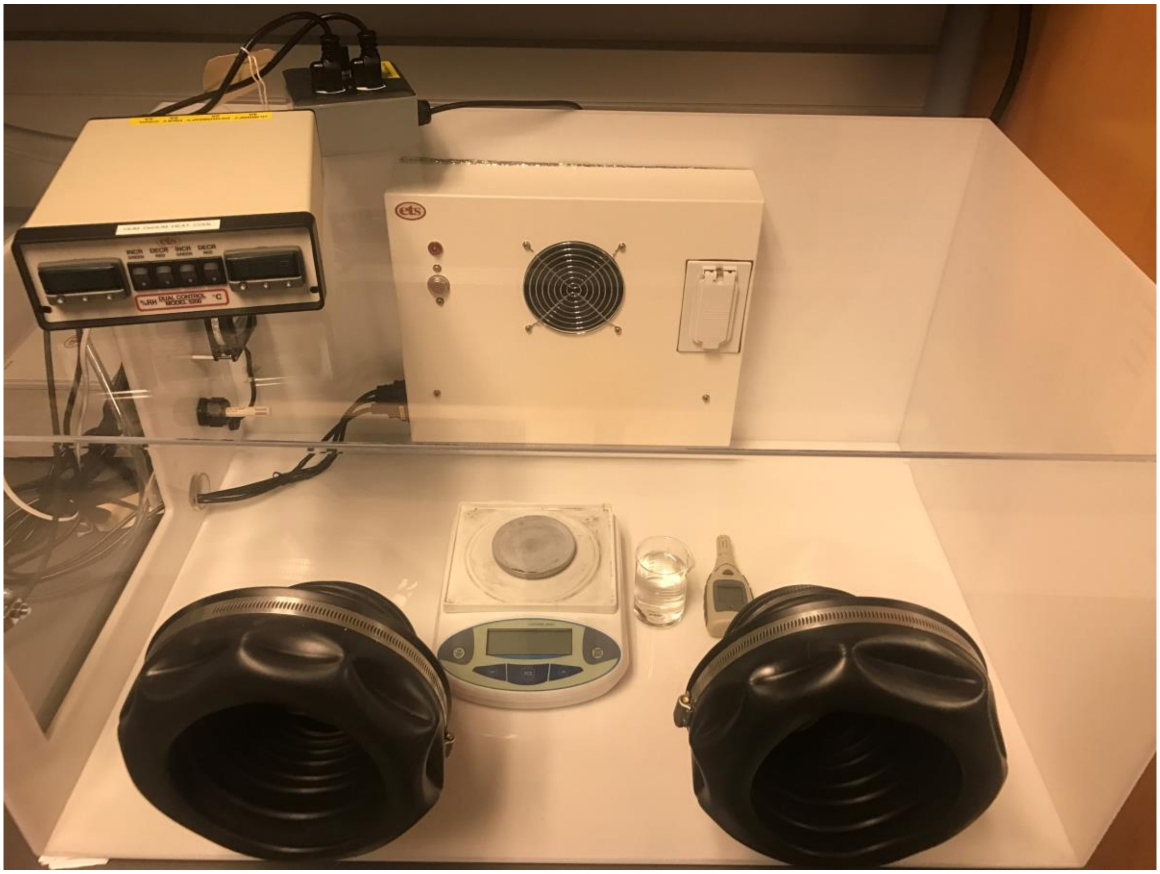

Fig. S2. Characterization of dark evaporation in controlled environment. The commercial glove box is 61 cm × 46 cm × 38 cm with controlled relative humidity and temperature inside the box.

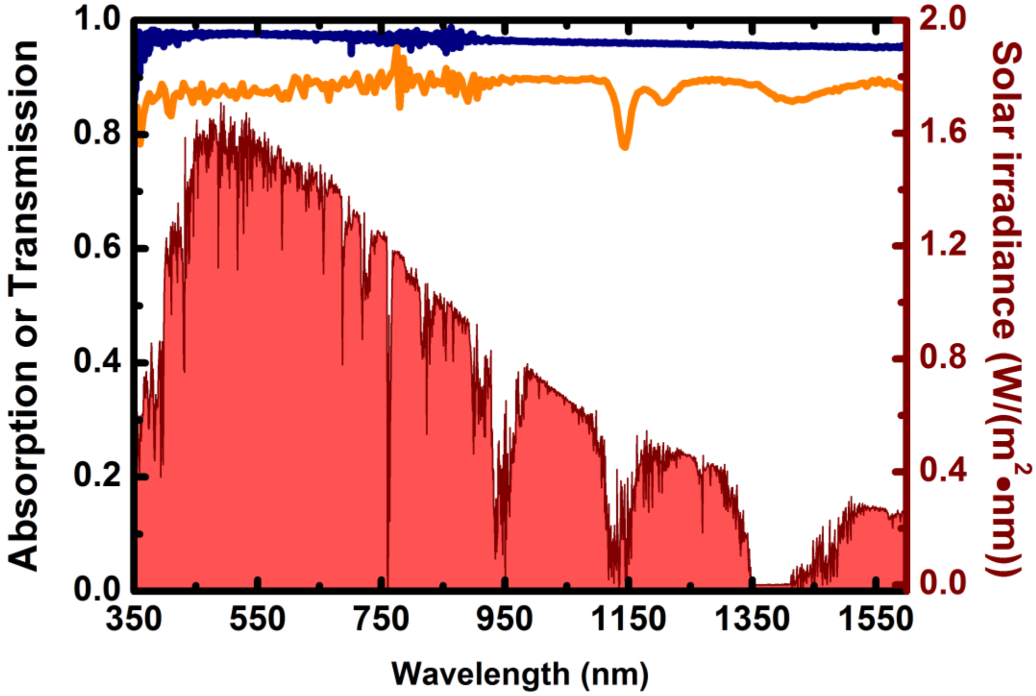

Fig. S3. The optical absorption spectrum of the CP and the transmission spectrum of the diffuser. The absorption is ~96.9% by weighting absorption spectrum (blue curve) with the AM 1.5 solar irradiance, which contributes to a high energy conversion efficiency. The red area shows the solar irradiation spectrum as a reference. The transmission spectrum (orange curve) indicates that the diffusers did not change the spectral feature of the incident light.

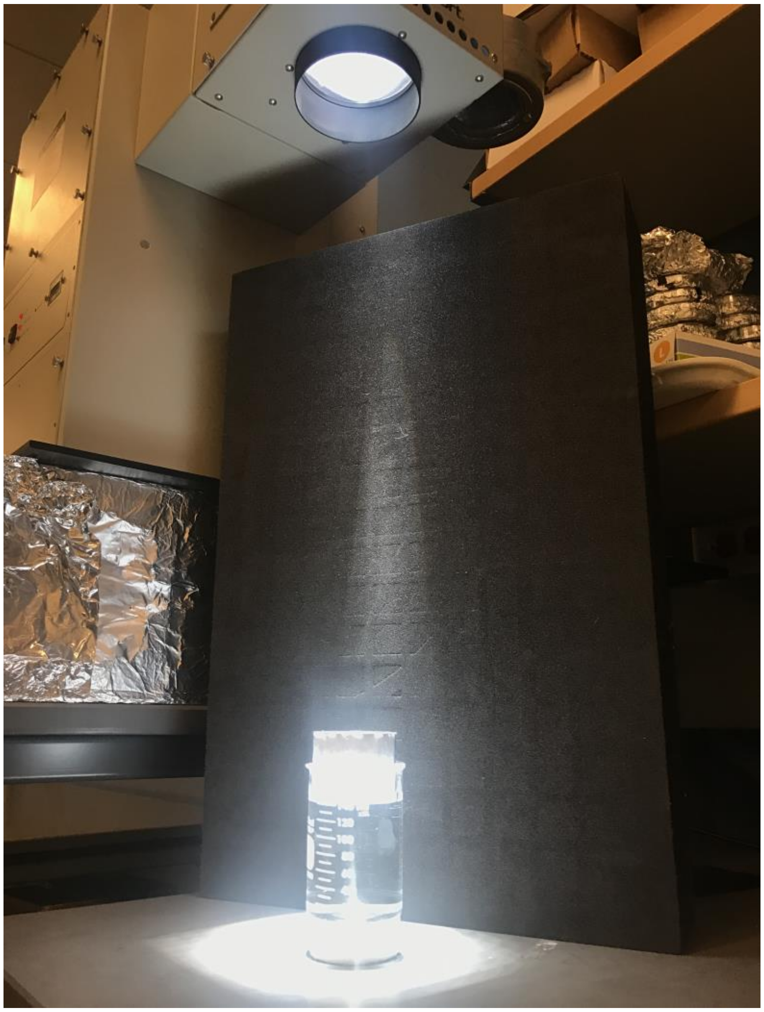

Fig. S4. The experimental setup for solar vapor generation. CP-foam is illuminated using the solar simulator.

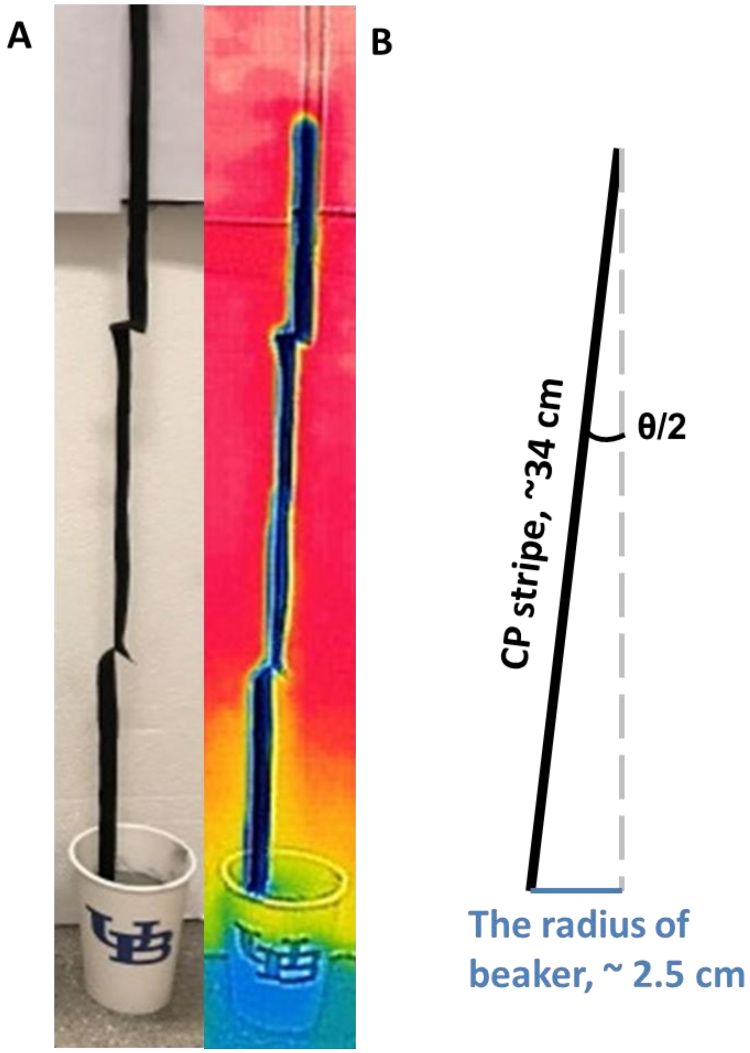

Fig. S5. The water transportation limit of the CP strip. (A)The thermal image of the CP with one end immersed in water. As the water evaporates, the temperature of the CP is lower than the ambient. The water transportation height is ~34 cm. (B) The practical limit of apex angle (θ) is calculated by considering the transportation limit of the CP stripe (i.e., ~ 34 cm) and the radius of the beaker (i.e., ~2.5 cm). Therefore, $\theta = 2 \times \arcsin(34\ \text{cm}/2.5\ \text{cm}) = 8.4°$.

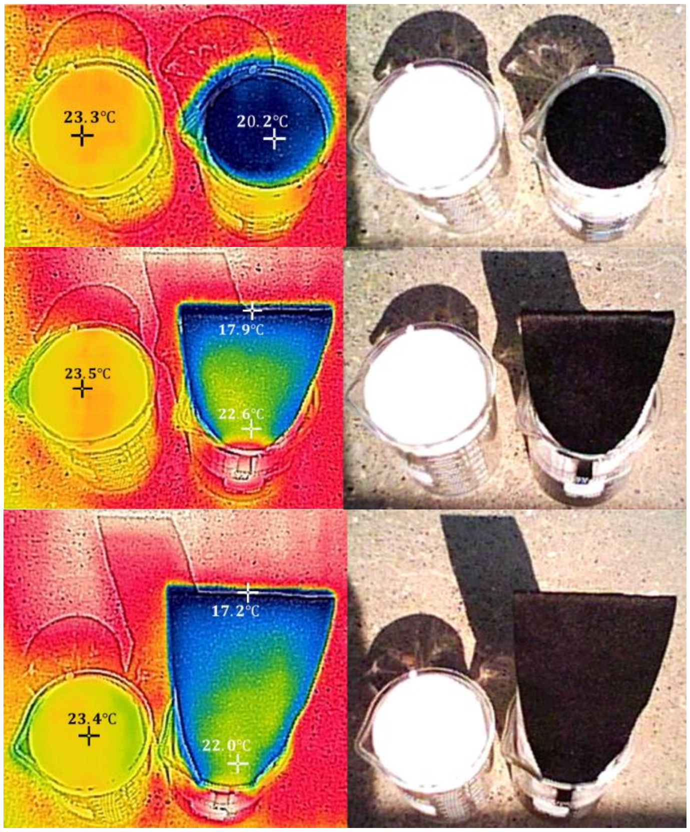

Fig. S6. Thermal distribution in an out-door environment. In this experiment, the solar intensity is ~0.267 sun with the room temperature and humility of ~23 ˚C and ~30%, respectively. Under uniform solar illumination, the temperature distribution of the CP surface is different from Fig. 4. To calibrate the temperature, a piece of white paper was adopted as a reference in the same thermal image. One can see that the temperature distribution is relatively uniform for a flat CP sample (upper panel), i.e., θ=180°. The temperature is slightly lower than the room temperature. When θ<180° (θ=37.8˚ in the middle panel and θ=22.9˚ in the lower panel), the temperature at the bottom of the CP is close to the room temperature, while the temperature at the top of the CP is only 17~18 ˚C. Therefore, the entire surface is below room temperature, producing cold vapor beyond the input solar limit. However, it is difficult to distinguish the contribution of the air flow in the out-door environment. Therefore, it is cleaner to claim the limit-breaking cold vapor generation in the laboratory environment, as we discussed in Fig. 4 of the main text.

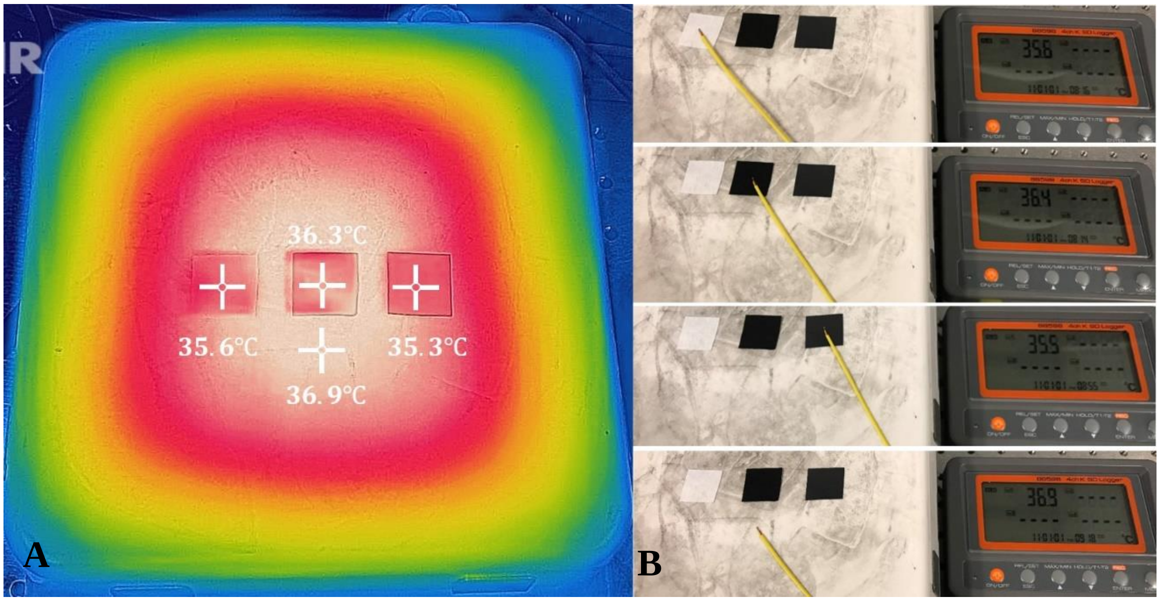

Fig. S7. Calibration of temperature measurement. (A) Surface temperature distribution of a white paper (left), a CP (central) and a black Al foil (right) placed on top of a heat plate set at 40 °C. (B) Direct measurement of the temperature at four positions using a thermal couple sensor probe.

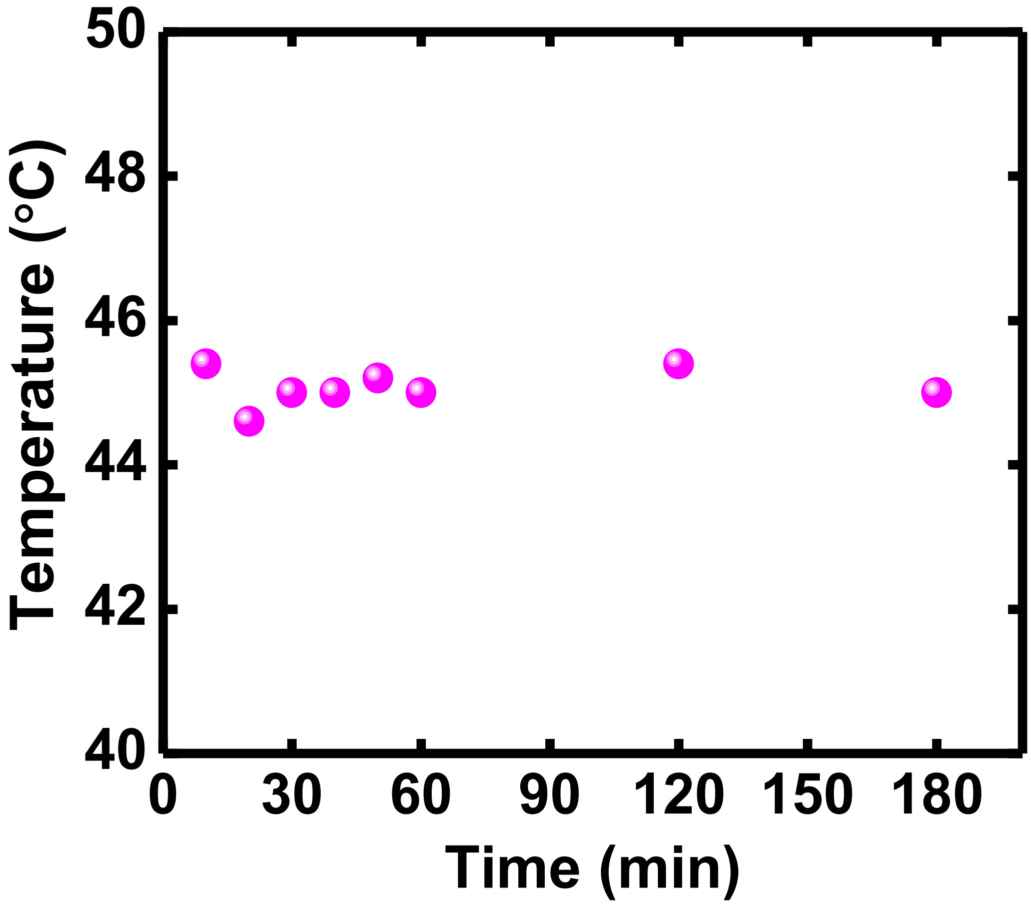

Fig. S8. Surface temperature change of the wet CP sample over three hours.

Table S1.

Measured dark evaporation rates of a bare water surface in controlled environment.

Relative humidity at the temperature of ~23.6 ºC	Rate ($kg\ m^{-2}\ h^{-1}$)	Temperature (ºC) at the relative humility of ~26%	Rate ($kg\ m^{-2}\ h^{-1}$)
26%±1%	0.0955	23±0.8	0.1009
46%±1%	0.0787	27±0.8	0.1070
66%±1%	0.0465	31±0.8	0.1315

Table S2.

Measured surface temperatures of a bare water surface and CP surface in controlled environment.

Relative humidity at the temperature of ~27.1 ºC	Bare water surface (ºC)	CP surface (ºC)
26%±1%	23.5	20.4
46%±1%	24.6	22.6
66%±1%	25.0	24.2

Table S3.
The values of solar intensity and the enthalpy of evaporation used in the calculation.

	Solar intensity (kW m^{-2})	Enthalpy of evaporation (J g^{-1})
Upper panel of Fig. 2B	0.609	2419.5
Lower panel of Fig. 2B	0.600	2435.7
Upper panel of Fig. 2D	0.203	2448.2
Lower panel of Fig. 2D	0.203	2453.6
Left panels of Fig. 4B	1.001	2399.9
Left panels of Fig. 4D	1.136	2433.9
Left panels of Fig. 4E	1.146	2439.1
Left panels of Fig. 4F	1.127	2437.1
Left panels of Fig. 4G	1.181	2444.2

Movie S1.

The surface temperature change of the CP after the solar light was turned off at the 4th second of the video.

Movie S2.

The surface temperature change of the CP when the solar light was turned on at the 5th second of the video.

Movie S3.

The surface temperature change of the CP in Fig. 4G when the solar light was turned on at the 3rd second of the video.

References

1 G. Crossmon, *The Anatomical Record*, 1937, **1**, 33-38.

2 D. G. Dortmund Data Bank Software & Separation Technology, Oldenburg, 2016.
